# Nationwide Heart Failure Telemonitoring: Disparities in Enrollment Based on Sex

**DOI:** 10.1016/j.jacadv.2025.102410

**Published:** 2025-12-04

**Authors:** Charell Jansen, Luuk C. Otterspoor, Petra van Pol, George C. Ijff, Steef J. Sinkeler, Ivo A. Joosen, Nienke Hermanides, Stefan Heinen, Mark J. Schuuring, Gerardus P.J. van Hout

**Affiliations:** a Department of Biomedical Signals and Systems, University of Twente, Enschede, The Netherlands; b Department of Cardiology, Medisch Spectum Twente, Enschede, the Netherlands; c Department of Cardiology, Catharina Hospital, Eindhoven, the Netherlands; d Department of Cardiology, OLVG, Amsterdam, the Netherlands; e Department of Cardiology, Maasstad Hospital, Rotterdam, the Netherlands; f Department of Cardiology, Martini Hospital, Groningen, the Netherlands; g Department of Cardiology, Canisius - Wilhelmina Hospital, Nijmegen, the Netherlands; h E-nurse, St. Antonius Hospital, Nieuwegein, the Netherlands; i Santeon, Utrecht, the Netherlands; j Department of Cardiology, St. Antonius Hospital, Nieuwegein, the Netherlands

**Keywords:** heart failure, home telemonitoring system (hTMS), eHealth, sex, gender

**What is the clinical question being addressed?**

Does sex-based disparity exist in enrollment in a nationwide heart failure telemonitoring program?

**What is the main finding?**

Women are significantly under-represented in heart failure telemonitoring enrollment, highlighting an urgent need for action to ensure equitable access and participation across sexes.

Home telemonitoring systems (hTMS) may be considered to reduce heart failure (HF) hospitalization and mortality rates according to the 2022 American Heart Association/American College of Cardiology/Heart Failure Society of America guideline for the Management of HF and 2021 European Society of Cardiology guideline on the diagnosis and treatment of HF.^1^^,^^2^ Furthermore, a meta-analysis from 2023 was an advocacy for the use of hTMS in HF patients to reduce all-cause mortality and HF-related hospitalizations.^3^

Despite growing evidence, there is a clear gap in the implementation and adoption of hTMS in clinical practice. The currently existing programs are highly heterogeneous.^3^ Therefore, a nationwide, reimbursed program was launched to standardize the hTMS and ensure uniform adoption. hTMS may be particularly effective during the medication optimization phase of the chronic HF care pathway, as many patients with HF receive suboptimal guideline-directed medical therapy in clinical practice. By remotely monitoring the process of medication optimization, hTMS is expected to help bridge this gap to achieve more optimal treatment goals.^4^ Ensuring equitable access is critical in chronic disease management.^5^ Therefore, we aimed to investigate gender differences to identify potential disparities and identify actionable opportunities in this real world hTMS program.

## Methods

The nationwide reimbursed hTMS program included standardized protocols for remote monitoring and interventions. All patients with symptomatic HF and reduced, mildly reduced, or preserved ejection fraction, irrespective of the etiology, were eligible. Monitoring involves patients measuring and recording their own blood pressure, heart rate, and weight, and reporting whether they experience symptoms or not. Values outside the patients specific thresholds result in an alarm to a local medical service center. Where trained nurses evaluate the incoming alerts, assisted by the treating clinician if needed. Standardized protocols include patient contact by phone, consults with the treating clinician and remote guideline-directed medical therapy optimization.

A national mutual software platform was used to export and integrate the data for analysis. Data were analyzed using IBM SPSS Statistics for Windows (version 29, IBM Corp.) and RStudio (version 4.4.1, RStudio Team). Data were not normally distributed. Therefore the Wilcoxon rank-sum test was used to compare groups.” A *P* value below 0.05 was considered statistically significant. No adjustments were made for potential confounders. All patient data were handled in accordance with applicable data protection and privacy regulations. The ethics committee of Santeon approved the study.

## Results

**Figure 1:**
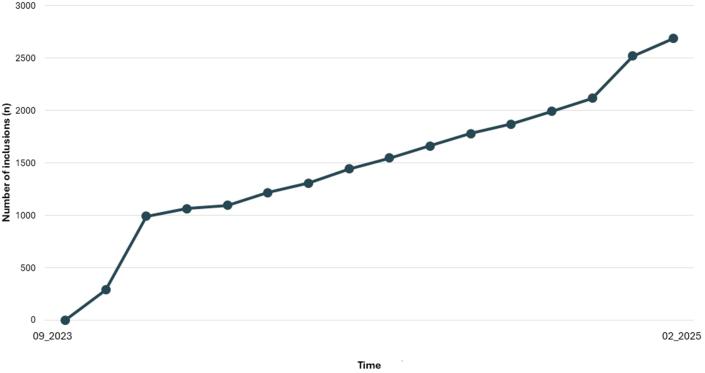
Enrollment in the Standardized Nationwide Home Telemonitoring Program This figure illustrates patient enrollment trends in the nationwide standardized home telemonitoring program for patients with heart failure. Data are presented across the study period, highlighting overall participation rates.

Seven large teaching hospitals, together the Santeon Network, in the Netherlands participate in the program. In September 2023, enrollment started, and after 17 months, 2,916 adult HF patients (2,041 [70%] men and 875 [30%] women) have been enrolled (Figure 1A). The figure shows a steep rise after launch of the program, followed by a plateau phase. After a year, another sharp increase occurs. Occasional declines appear due to participants stopping the monitoring for various reasons, including mortality. Despite fluctuations, the long-term inclusion rates remain on an upward trend.

In the overall cohort, the mean age was 70 ± 11 years for men and 71 ± 11 years for women (*P* < 0.001). Participants in the hTMS program were slightly younger, with a mean age of 67 ± 11 years, compared to 71 ± 11 years for nonparticipants (*P* < 0.001). For participants in the standardized hTMS group, the mean age was equal for men and women (68 vs 67 years).

**Figure 2:**
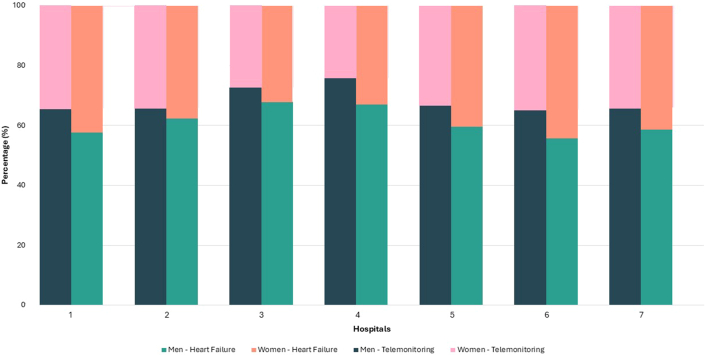
Heart Failure and Home Telemonitoring System Use: Men Vs Women This figure illustrates the distribution by sex and hospital. Per bar the number and percentages of men and women in the cohort are shown. The figure emphasizes observed disparities in enrollment between male and woman patients, which may reflect referral patterns. These findings underscore the importance of addressing equity in program implementation to ensure broader, more inclusive adoption of telemonitoring in heart failure care.

In the overall cohort from 7 hospitals, HF was diagnosed more often in men (n = 7,125; 62%) than in women (n = 4,426; 38%). There were significantly higher hTMS participation rates among men (29% (2,041/7,125) compared to women (20% (875/4,426), *P* < 0.001) (Figure 2).

## Discussion

Rapid upscaling is feasible with the use of a nationwide standardized hTMS program. Participants of the hTMS program were slightly younger compared to nonparticipants. Within the analyzed HF population, a disproportional high participation rate of men was observed compared to women, whereas both groups had comparable ages from a clinical standpoint. The exact mechanism for enrollment disparities is unclear. Possible factors include referral patterns, digital literacy, and gender-specific attitudes toward technology. Socioeconomic status, caregiver support, and cultural expectations may also contribute. These influences highlight the need for additional research and both strategies and interventions to improve equitable hTMS access and uptake. Further research and increased awareness are crucial to maximize the program's effectiveness, particularly for women with HF. Examining how eHealth solutions like hTMS are adopted is essential for implementing novel technologies in routine care. The rapid increase of participants of this hTMS revealed the gaps and the readiness of the population to adopt these solutions. To prevent disparities, eHealth solutions should be accessible to all HF patients. Targeted outreach, digital literacy support, and gender-sensitive design may improve equitable hTMS access, particularly among women.

## Funding support and author disclosures

The national program was funded by insurance companies and was supported by the national health transformation fund. Research was funded by Stichting Hartcentrum Twente (Twente Heart Center Foundation). Dr Schuuring acknowledges being a member of a Dutch CardioVascular Alliance consortium (ADMINISTER II), which is supported by public/not-for-profit organizations (i.e. Stichting Hartcentrum Twente) and partners with independent contributions to the research institute (10.13039/100004325AstraZeneca & 10.13039/100001003Boehringer Ingelheim) and also received grant funding from the Pioneers in Healthcare scheme (10.13039/501100001834University of Twente) and TKI-PPP (Health Holland). Dr van Hout received a grant by the St. Antonius research funds to pursue scientific research on the hTMS system in heart failure. All other authors have reported that they have no relationships relevant to the contents of this paper to disclose.
